# Deregulation of the actin cytoskeleton and macropinocytosis in response to phorbol ester by the mutant protein kinase C gamma that causes spinocerebellar ataxia type 14

**DOI:** 10.3389/fphys.2014.00126

**Published:** 2014-04-01

**Authors:** Kazuhiro Yamamoto, Takahiro Seki, Hikaru Yamamoto, Naoko Adachi, Shigeru Tanaka, Izumi Hide, Naoaki Saito, Norio Sakai

**Affiliations:** ^1^Department of Molecular and Pharmacological Neuroscience, Graduate School of Biomedical and Health Sciences, Hiroshima UniversityHiroshima, Japan; ^2^Department of Chemico-Pharmacological Sciences, Graduate School of Pharmaceutical Sciences, Kumamoto UniversityKumamoto, Japan; ^3^Biosignal Research Center, Kobe UniversityKobe, Japan

**Keywords:** spinocerebellar ataxia type 14, γPKC, translocation, actin cytoskeleton, macropinocytosis, MARCKS

## Abstract

Several missense mutations in the protein kinase C**γ** (**γ**PKC) gene have been found to cause spinocerebellar ataxia type 14 (SCA14), an autosomal dominant neurodegenerative disease. γPKC is a neuron-specific member of the classical PKCs and is activated and translocated to subcellular regions as a result of various stimuli, including diacylglycerol synthesis, increased intracellular Ca^2+^ and phorbol esters. We investigated whether SCA14 mutations affect the γPKC-related functions by stimulating HeLa cells with TPA (12-O-tetradecanoylpholbol 13-acetate), a type of phorbol ester. Wild-type (WT) γPKC-GFP was translocated to the plasma membrane within 10 min of TPA stimulation, followed by its perinuclear translocation and cell shrinkage, in a PKC kinase activity- and microtubule-dependent manner. On the other hand, although SCA14 mutant γPKC-GFP exhibited a similar translocation to the plasma membrane, the subsequent perinuclear translocation and cell shrinkage were significantly impaired in response to TPA. Translocated WT γPKC colocalized with F-actin and formed large vesicular structures in the perinuclear region. The uptake of FITC-dextran, a marker of macropinocytosis, was promoted by TPA stimulation in cells expressing WT γPKC, and FITC-dextran was surrounded by γPKC-positive vesicles. Moreover, TPA induced the phosphorylation of MARCKS, which is a membrane-substrate of PKC, resulting in the translocation of phosphorylated MARCKS to the perinuclear region, suggesting that TPA induces macropinocytosis via γPKC activation. However, TPA failed to activate macropinocytosis and trigger the translocation of phosphorylated MARCKS in cells expressing the SCA14 mutant γPKC. These findings suggest that γPKC is involved in the regulation of the actin cytoskeleton and macropinocytosis in HeLa cells, while SCA14 mutant γPKC fails to regulate these processes due to its reduced kinase activity at the plasma membrane. This property might be involved in pathogenesis of SCA14.

## Introduction

Protein kinase C (PKC), a member of the serine/threonine kinase family, has been implicated in signal transduction and the regulation of various cellular functions (Rosse et al., 2010). PKC isoforms have been classified into three groups, classical PKCs (cPKC: α, β I, β II, and γ), novel PKCs (nPKC: δ, ε, η, and θ) and atypical PKCs (aPKC: ι/λ and ζ), according to their different structures and activators. cPKCs have a C1 domain that binds to diacylglycerol (DG) and phorbol esters, well-known PKC activators, and a Ca^2+^-binding C2 domain in its regulatory subunit (Shirai and Saito, 2002). We have previously performed live imaging studies using green fluorescent protein (GFP)-tagged PKC (PKC-GFP), and we demonstrated that PKCs are translocated to several cellular organelles in a subtype- and stimulation-specific manner as a result of activation by various stimuli (Sakai et al., 1997; Shirai et al., 1998). Thereafter, PKCs recognize and phosphorylate their substrates in the targeted subcellular regions, resulting in the subsequent cellular responses (i.e., PKC targeting).

Spinocerebellar ataxia type 14 (SCA14) is an autosomal dominant neurodegenerative disorder clinically characterized by progressive ataxia due to Purkinje cell (PC) degeneration (Brkanac et al., 2002; Chen et al., 2003). To date, 28 missense and deletion mutations in the *PRKCG* gene, which encodes protein kinase Cγ (γPKC), have been identified as the cause of SCA14 (Chen et al., 2005, 2012; Hiramoto et al., 2006). Among the PKC subtypes, γPKC is specifically expressed in the central nervous system and is especially abundant in cerebellar PCs (Saito et al., 1988). We previously reported that mutant γPKCs tend to aggregate in cells in culture (Seki et al., 2005a, 2011), causing apoptotic cell death via the impairment of the ubiquitin proteasome system and the induction of ER stress (Seki et al., 2007). Furthermore, we have reported that mutant γPKC induces the improper development of dendrites in cultured PCs, regardless of the presence or absence of its aggregates (Seki et al., 2009). This finding raises the possibility that mutant γPKC might disturb the regulation of the cytoskeleton, which is important for neuritic development (Meyer and Feldman, 2002), independently of aggregate formation.

Additionally, the growth, remodeling and maintenance of the axonal and dendritic processes of neurons largely depend on membrane trafficking. Membrane vesicles, referred to as cargos, transport a set of molecules, such as proteins and lipids, within a cell. Emerging evidence has shown that vesicular cargos are compartmentalized in neuron- and neurite-specific manners and play important roles in neurite outgrowth, guidance and maintenance (Horton and Ehlers, 2004; Hanus and Ehlers, 2008; Sann et al., 2009). Endocytosis is a mechanism that results in the internalization of membranes from the plasma membrane lipid bilayer. During this process, the plasma membrane lipids, integral proteins and extracellular fluid become fully internalized into the cell. Endocytosis also regulates the lipid and protein composition of the plasma membrane (Doherty and McMahon, 2009). Accumulating evidence has demonstrated the involvement of PKC in membrane trafficking and endocytosis (Aballay et al., 1999; Song et al., 1999; Prevostel et al., 2000; Alvi et al., 2007; Abreu et al., 2008). Therefore, it is possible that γPKC participates in the regulation of membrane trafficking and endocytosis in PCs. This finding led to the hypothesis that SCA14 mutations disturb this regulation, resulting in the aberrant morphology of the PC dendrites. In the present study, we demonstrate that γPKC plays a role in the regulation of membrane trafficking, endocytosis (macropinocytosis) and the actin cytoskeleton in HeLa cells, while SCA14 mutant γPKC fails to regulate these processes due to its reduced phosphorylation of its membrane substrate.

## Materials and methods

### Materials

The anti-GFP mouse monoclonal antibody and nocodazole were purchased from Nakalai Tesque (Kyoto, Japan). The anti-β -tubulin 1, anti-γ-tubulin and anti-ubiquitin mouse monoclonal antibodies, 12-*O*-Tetradecanoylphorbol 13-acetate (TPA), nocodazole and FITC-conjugated dextran 70 kDa were obtained from Sigma-Aldrich. The anti-γPKC rabbit polyclonal antibodies were purchased from Santa Cruz Biotechnology (Santa Cruz, CA, USA). The Alexa 546-conjugated secondary antibodies and phalloidin-TRITC were obtained from Invitrogen. The plasmid encoding YFP-mem was obtained from Clontech. The anti-p62 guinea pig polyclonal antibody was purchased from PROGEN biotechnik GmbH. Horseradish peroxidase (HRP)-conjugated secondary antibodies for western blot analysis were purchased from Jackson ImmunoResearch Laboratories (West Grove, PA, USA). The anti-p-MARCKS rabbit polyclonal antibody was a gift from Dr. Hideyuki Yamamoto, Ryukyu University, Japan (Yamamoto et al., 1998).

### Cell culture and transfection

HeLa cells were cultured in DMEM in a humidified atmosphere containing 5% CO_2_ at 37°C. The medium was supplemented with 10% fetal bovine serum, 100 units/ml penicillin and 100 μg/ml streptomycin.

Mouse cerebellar primary culture was prepared as described previously (Seki et al., 2009). Briefly, E14 embryos from pregnant ICR mice were dissociated with the dissociation solutions of the SUMITOMO Nerve-Cell Culture System according to the manufacturer's protocol. Dissociated cerebellar cells were cultured on a 3.5-cm glass-bottom dish (MatTek, Ashland, MA, USA) using the neuron culture medium of the SUMITOMO Nerve-Cell Culture System. Cells were cultured for 28 days *in vitro* (DIV) in a humidified atmosphere containing 5% CO_2_.

### Expression of γPKC-GFP/γPKC-HT in HeLa cells and cultured Purkinje cells

We previously characterized the molecular properties of various SCA14 mutant γPKCs (Seki et al., 2005a, 2011). Among these mutants, we selected two mutants (S119P and G128D) that are highly aggregate-prone and cytotoxic, and one mutant (S119F) that does not form aggregates but is cytotoxic to analyze in the present study.

WT and mutant (S119P, G128D, and S119F) γPKC-GFP or γPKC-HaloTag (HT) was expressed in HeLa cells using adenoviral vectors, as previously described (Yamamoto et al., 2010). Briefly, the cells were infected with two types of adenoviral vectors, Ad-CMV-tTA and Ad-TetOp-γPKC-GFP/Ad-TetOp-γPKC-HT at an MOI of 10. These adenoviral vectors were constructed as previously described (Seki et al., 2009).

To express MARCKS-GFP and YFP-mem in HeLa cells, the plasmids were transfected using the FuGENE6 transfection reagent (Roche diagnostics), according to the manufacturer's protocol.

### Fluorescence imaging

Transfected HeLa cells were cultured on a 3.5-cm glass-bottom dish for 24 h. Time-lapse imaging of γPKC-GFP was conducted using a BIOREVO (Keyence, Osaka, Japan) fluorescence microscope, with a stage-top CO_2_ incubator (Tokai-Hit, Fujinomiya, Japan). Sequential GFP fluorescence images were obtained every 1 min for 1 h after treatment with 100 nM TPA.

HeLa cells expressing γPKC-GFP were treated with 100 nM TPA for 15, 30, and 60 min, followed by fixation with 4% paraformaldehyde and 0.2% picric acid. Nocodazole (1 μg/ml) and Gö6976 (2.5 μg/ml) were added 20 min prior to TPA treatment. Immunofluorescence and F-actin staining were conducted as previously described (Seki et al., 2013). Briefly, after the membrane was permeabilized with 0.3% Triton X100, the fixed cells were incubated with 100 nM phalloidin-TRITC to stain the F-actin. For immunofluorescence staining, the permeabilized cells were incubated with the anti-γ-tubulin antibody (1:500) or anti-phosphorylated MARCKS antibody (1:200), followed by incubation with an Alexa 568-conjugated secondary antibody (1:500). After washing, fluorescence and differential interference contrast (DIC) images were obtained using a LSM5 PASCAL confocal laser scanning microscope (Carl Zeiss). The sizes of γPKC-GFP-expressing cells were calculated from GFP-fluorescence images by Image-Pro Plus 5.1 (Media Cybernetics, Bethesda, MD, USA).

### Uptake of macropinocytosis marker

Cells expressing γPKC-HT were incubated in the presence of 50 nM tetramethylrhodamine (TMR)-conjugated HT ligand (Promega) for 24 h to label the γPKC-HT with TMR. The cells were incubated with 5 mg/ml FITC-dextran, a fluorescent marker of macropinocytosis, in the presence or absence of 100 nM TPA for 30 min. After fixation, fluorescence and DIC images were obtained using a confocal laser scanning microscope.

### Statistical analysis

Unpaired Student's *t*-test was used to determine statistical differences between two groups indicated in figure legends. Prism 4 software (GraphPad Software, La Jolla, CA) was used to conduct Student's *t*-test and to generate graphs.

## Results

### Phorbol ester results in the translocation of γPKC-GFP to the Perinuclear region and induces cell shrinkage

**Figure 1:**
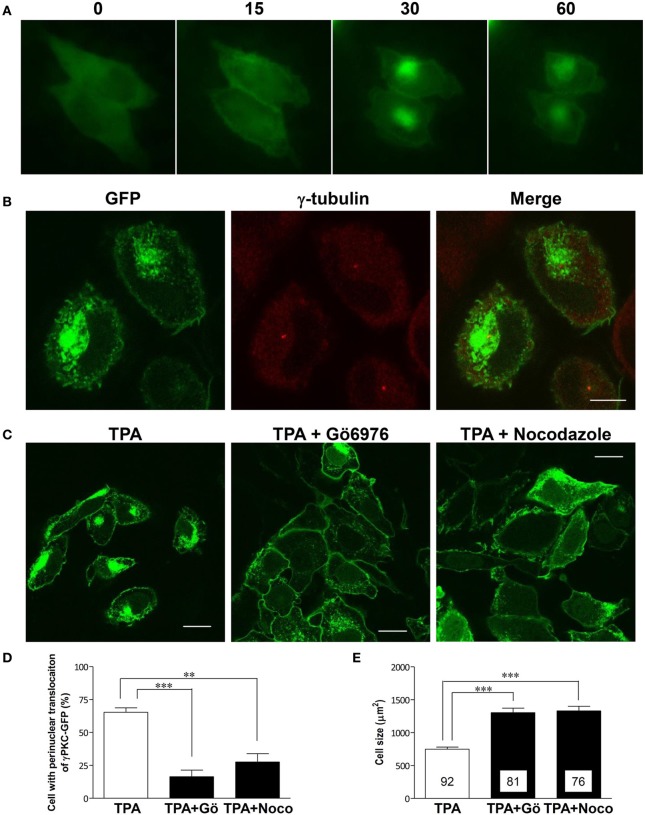
**γ PKC is translocated around the centrosome in microtubule- and PKC kinase activity-dependent manners**. **(A)** Time-lapse imaging of γPKC-GFP expressing HeLa cells treated with TPA. γPKC-GFP expressing cells were treated with 100 nM TPA for 1 h. These images were obtained from the same cells at each time point (0, 15, 30, and 60 min) after TPA treatment. The translocations of γPKC-GFP to the plasma membrane (15 min) and to the perinuclear region (30 and 60 min) were observed, accompanied by a reduction in cell size (30 and 60 min). Bar = 10 μm. **(B)** γPKC translocation around the centrosome. Representative fluorescence images (γPKC-GFP, γ-tubulin, and merged) of γPKC-GFP-expressing HeLa cells treated with 100 nM TPA for 60 min, followed by immunostaining with an anti-γ-tubulin antibody, a marker of the centrosome. Translocated γPKC-GFP was localized around the centrosome. Bar = 10 μm. **(C–E)** γPKC translocation around the centrosome and cell shrinkage were dependent on microtubules and PKC kinase activity. **(C)** Representative fluorescence images of γPKC-GFP-expressing cells treated with TPA alone (left), TPA plus 1 μg/ml nocodazole, an inhibitor of tubulin polymerization (center) and TPA plus 2.5 μg/ml Gö6976, an inhibitor of cPKC (right). Nocodazole and Gö6976 were added 20 min prior to treatment with 100 nM TPA. The cells were fixed 30 min after TPA treatment. Nocodazole and Gö6976 prevented the TPA-induced perinuclear translocation of γPKC-GFP and cell shrinkage. Bar = 10 μm. **(D)** The percentage of γPKC-GFP-positive HeLa cells with perinuclear translocation after 30 min of TPA treatment, shown in **(C)**. The data represent the mean ± standard error from four independent experiments. **(E)** The cell size of the GFP-positive HeLa cells after TPA treatment for 30 min, shown in **(C)**. The data represent the mean ± standard error. The number of quantified cells in each treatment is indicated in the column. ^**^p < 0.01, ^***^p < 0.001 (unpaired *t*-test).

Phorbol ester activates PKC by binding to its C1 domain, accompanied by its translocation to the plasma membrane within 10 min (Sakai et al., 1997). However, prolonged treatment with phorbol ester induces the subsequent translocation of α and β PKC to the perinuclear region (Becker and Hannun, 2003). To observe these phenomena in the same cells expressing γPKC, time-lapse observations were conducted in HeLa cells expressing γPKC-GFP, which were treated with 100 nM TPA (12-O-tetradecanoylphorbol-13-acetate) for 1 h. TPA treatment resulted in the translocation of γPKC-GFP from the cytoplasm to the plasma membrane within 15 min, followed by its accumulation in the perinuclear region (Figure 1A) and cell shrinkage within 30–60 min (Supplemental Figure 1). Perinuclear accumulated γPKC-GFP was localized around the centrosome (Figure 1B). This accumulation and the cell shrinkage were prevented by nocodazole-mediated microtubule depolymerization and the Gö6976-mediated inhibition of PKC kinase activity (Figures 1C–E). These findings suggest that the perinuclear translocation of γPKC and the cell shrinkage are dependent on the microtubules and PKC kinase activity. A similar perinuclear translocation, but not cell shrinkage, was observed 60 min after TPA treatment in primary cultured PCs expressing γPKC-GFP (Supplemental Figure 2)

### SCA14 mutant γPKCs fail to translocate to the perinuclear region and to induce cell shrinkage in response to TPA treatment

**Figure 2:**
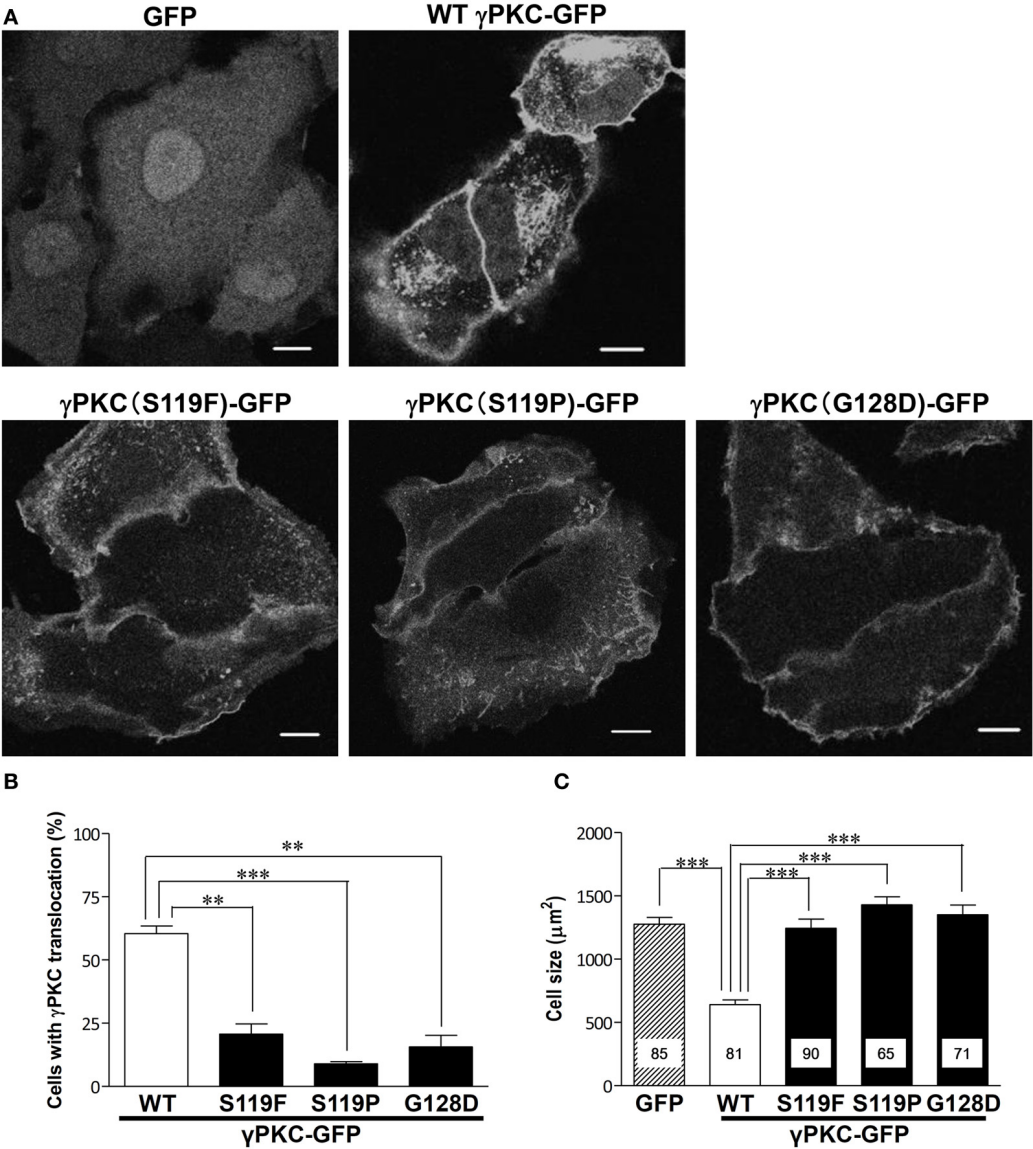
**SCA14 mutant γ PKC impairs the perinuclear translocation and cell size reduction in response to TPA treatment. (A)** Representative fluorescence images of GFP (left), WT γPKC-GFP (center) or S119P γPKC-GFP (right)-expressing HeLa cells treated with TPA for 30 min. In response to TPA treatment for 30 min, WT γPKC-GFP exhibited membrane translocation, followed by perinuclear translocation and a reduction in cell size. In contrast, S119P mutant γPKC-GFP did not exhibit perinuclear translocation and remained at the plasma membrane. In cells expressing GFP alone, TPA did not induce the translocation of GFP or cell shrinkage. Scale bar = 10 μm. **(B, C)** Quantification of the TPA-induced perinuclear translocation of γPKC-GFP and cell shrinkage. **(B)** The percentage of γPKC-GFP-positive HeLa cells with perinuclear translocation, shown in **(A)**. WT and mutant (S119F, S119P, and G128D) γPKC-GFP were expressed in HeLa cells and were stimulated with TPA for 30 min. After fixation, the percentage of cells with perinuclear translocation was evaluated. The data represent the mean ± standard error from three independent experiments. **(C)** The cell size of the GFP-positive HeLa cells after TPA treatment, shown in **(A)**. GFP, WT, and mutant γPKC-GFP were expressed in HeLa cells and were stimulated with TPA for 30 min. After fixation, the cell size was measured from the GFP fluorescence images. The data represent the mean ± standard error. The number of quantified cells in each treatment is indicated in the column. ^**^p < 0.01, ^***^p < 0.001 (unpaired *t*-test).

Next, we examined whether the SCA14 mutations affects these TPA-triggered phenomena. HeLa cells expressing GFP, wild-type (WT) or S119P mutant γPKC-GFP were treated with 100 nM TPA. TPA treatment did not induce cell shrinkage in the cells expressing GFP alone, while in the WT γPKC-GFP cells, it triggered the perinuclear translocation of γPKC-GFP and induced cell shrinkage (Figure 2A), as described above. On the other hand, TPA induced the initial translocation of the S119P mutant γPKC-GFP to the plasma membrane, and mutant γPKC-GFP remained at the plasma membrane 30 min after TPA treatment (Figure 2A). The perinuclear translocation and cell shrinkage after TPA treatment were significantly impaired in the cells expressing three different mutant γPKC-GFPs (S119F, S119P, and G128D) (Figures 2B,C). We have previously reported that mutant γPKCs tend to form aggregates in the perinuclear region (Seki et al., 2005a). Although mutant γPKC-GFP aggregates were colocalized with ubiquitin and p62, the perinuclear WT γPKC-GFP was not colocalized with these signals (Supplemental Figure 3), indicating that the perinuclear translocation of WT γPKC is a different cellular event from the aggregation of mutant γPKC.

### Phorbol ester induces the remodeling of the actin cytoskeleton and macropinocytosis via the activation of γPKC, but not the SCA14 mutant γPKCs

**Figure 3:**
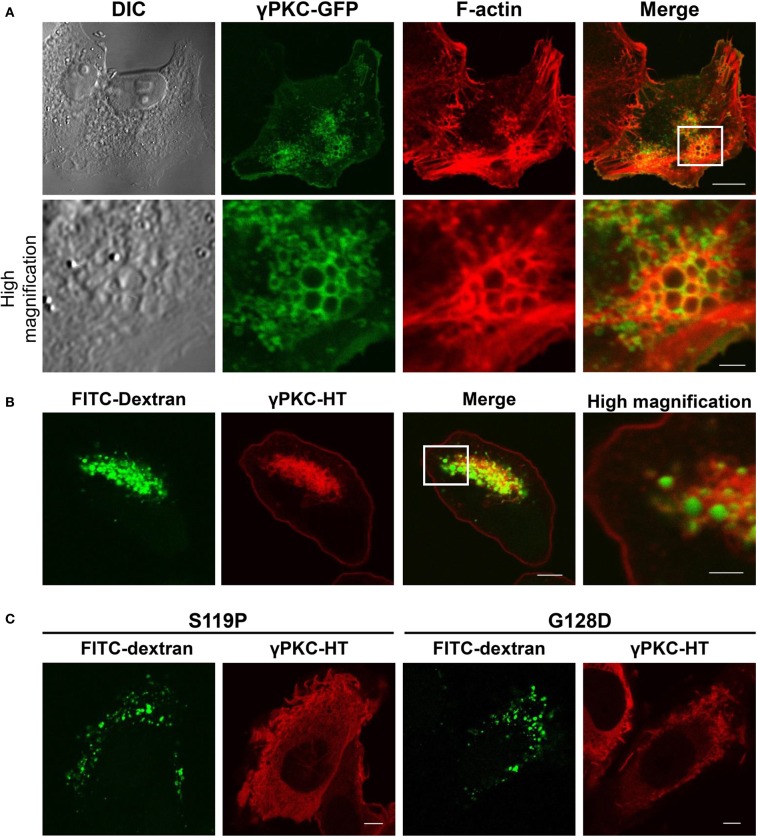
**γ PKC, but not SCA14 mutant γ PKC, mediates the TPA-induced remodeling of the actin cytoskeleton and the activation of macropinocytosis**. **(A)** Representative images (DIC, γPKC-GFP, F-actin, and merged) of γPKC-GFP-expressing HeLa cells treated with TPA for 30 min. The lower panels show high magnification images of the boxed areas in the merged images in the upper panels. F-actin was visualized by staining with 100 nM phalloidin-TRITC. Perinuclear γPKC-GFP was colocalized with F-actin around many large vesicles (> 0.2 μm in a diameter). The scale bars in the upper and lower panels are 10 and 2 μm, respectively. **(B)** FITC-dextran uptake in γPKC-HT-expressing cells in response to TPA treatment. FITC-dextran, a fluorescent marker for macropinocytosis, was simultaneously incubated with cells expressing γPKC-HT for 30 min during TPA treatment. Representative images (γPKC-HT, FITC-dextran and merged) are shown. The far right image shows the high magnification image of the boxed area in the merged image. FITC-dextran was surrounded by γPKC-HT. The scale bars in the merged and high magnification images are 5 and 2 μm, respectively. **(C)** FITC-dextran uptake in WT and mutant (S119P and G128D) γPKC-HT-expressing cells in response to TPA treatment. Cells expressing γPKC-HT were simultaneously incubated for 30 min with FITC-dextran and TPA. Representative images (γPKC-HT and FITC-dextran) are shown. TPA enhanced the uptake of FITC-dextran in cells expressing WT γPKC-HT, but not in cells expressing mutant γPKC-HT. Scale bar = 5 μm.

Because morphological changes in cells commonly accompany the remarkable remodeling of the cytoskeleton, we stained the F-actin of the TPA-treated cells expressing WT γPKC-GFP with TRITC-phalloidin. F-actin was colocalized with WT γPKC-GFP, which formed vesicular structures with diameters of 0.2–1.5 μm (Figure 3A). These vesicles were also found in high-magnification images taken with a differential interference contrast (DIC) microscope (Figure 3A). The TPA-induced vesicular structures were colocalized with YFP-mem, a fluorescence marker of the plasma membrane (Supplemental Figure 4), indicating that these vesicles are endosomes that are derived from the plasma membrane. We next focused on the cellular mechanism by which these vesicles are formed from the plasma membrane. Macropinocytosis, which was first observed by Lewis (1931) and is important for the bulk and non-selective uptake of extracellular fluid, is actin-dependent and characterized by large macropinosomes (>0.2 μm in diameter) (Amyere et al., 2002; Lim and Gleeson, 2011). Therefore, we assumed that these endosomes were formed by the activation of macropinocytosis. To verify whether TPA induced macropinocytosis, we examined the uptake of FITC-dextran, a marker of the macropinosome (Lee and Knecht, 2002), in cells expressing γPKC-HaloTag (HT). HT is a unique protein that can be fluorescently labeled by the addition of the HT ligand fused to fluorescent dye to the culture medium (Seki et al., 2012), and γPKC-HT was labeled with tetramethylrhodamine (TMR) by incubating the cells with the TMR-conjugated HT ligand for 24 h. TPA induced the uptake of FITC-dextran, which was surrounded by γPKC-HT (Figure 3B). Additionally, we found that FITC-dextran was also taken up in the TPA-treated cells expressing αPKC and δ PKC (Supplemental Figure 5). Previously, it was reported that αPKC plays a role in clathrin-mediated endocytosis (CME) (Idkowiak-Baldys et al., 2006). To examine whether the γPKC-positive vesicles were derived from CME, the cells were immunostained with AP2α, a marker of clathrin-coated vesicles (Doherty and McMahon, 2009). Vesicles containing γPKC did not colocalize with AP2α immunoreactivity (Supplemental Figure 6). These findings indicate that TPA induces the remodeling of the actin cytoskeleton and macropinocytosis, but not CME, via the activation of γPKC. Similar to the perinuclear translocation and cell shrinkage, TPA failed to increase the uptake of FITC-dextran in cells expressing SCA14 mutant γPKC-HT (Figure 3C).

### MARCKS, a PKC substrate, is involved in the TPA-induced activation of macropinocytosis

**Figure 4:**
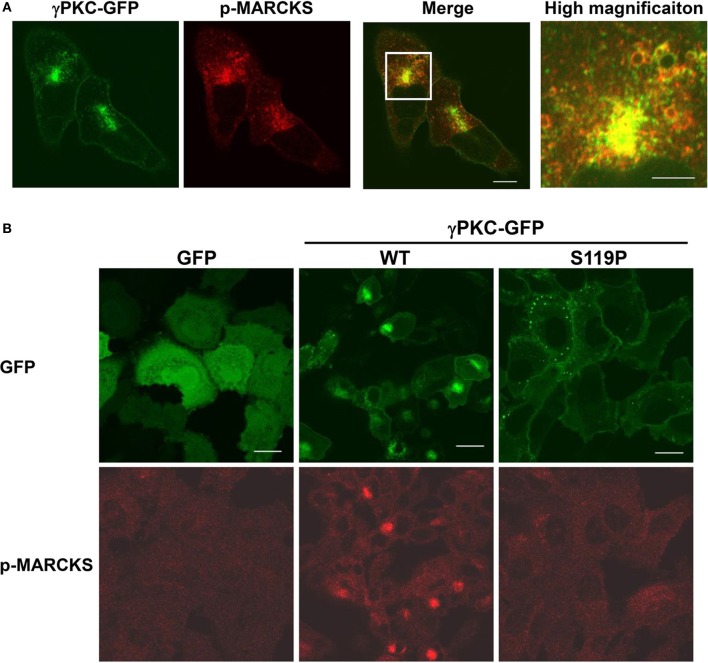
**γ PKC, but not SCA14 mutant γ PKC, mediates the TPA-induced perinuclear translocation of phosphorylated MARCKS. (A)** Perinuclear translocation of phosphorylated MARCKS (p-MARCKS) 30 min after TPA treatment. Representative images (γPKC-GFP, p-MARCKS and merged) are shown. Cells expressing γPKC-GFP were treated with TPA for 30 min, followed by immunostaining with an anti-p-MARCKS antibody. The far right image shows the high magnification image of the boxed area in the merged image. p-MARCKS was strongly colocalized with the perinuclear γPKC-GFP, especially around the vesicles. The scale bars of the merged and high magnification images are 5 and 2 μm, respectively. **(B)** Localization of p-MARCKS in cells expressing GFP alone, WT and S119P γPKC-GFP 30 min after TPA treatment. Representative images (p-MARCKS and GFP) are shown. As described above, p-MARCKS was colocalized with WT γPKC-GFP in the perinuclear region. In contrast, obvious p-MARCKS immunostaining was not observed in cells expressing GFP alone or mutant γPKC-GFP. Scale bar = 10 μm.

We attempted to determine which PKC substrate participates in these phenomena. We focused on MARCKS (myristoylated alanine-rich C-kinase substrate) because this molecule is a well-known PKC substrate that is located on the plasma membrane and is involved in regulation of the actin cytoskeleton (Li et al., 2008) and endocytosis (Carballo et al., 1999; Song et al., 1999). To investigate the involvement of MARCKS in remodeling of the actin cytoskeleton and macropinocytosis, the cells were immunostained with an antibody that specifically targeted phosphorylated MARCKS (p-MARCKS). We confirmed that TPA increased the level of p-MARCKS, especially in cells expressing WT γPKC-GFP, through immunoblot analysis (Supplemental Figure 7A). In response to TPA, p-MARCKS was colocalized with γPKC-GFP in the perinuclear region (Figure 4A), suggesting that the phosphorylation of MARCKS by γPKC might be involved in the remodeling of the actin cytoskeleton and the subsequent translocation of γPKC around the centrosome, along with F-actin and p-MARCKS. In contrast, TPA failed to translocate p-MARCKS in cells expressing SCA14 mutant γPKC-GFP, as well as in the GFP-expressing cells (Figure 4B). Indeed, TPA only slightly increased the phosphorylation of MARCKS in the cells expressing SCA14 mutant γPKC-GFP, equivalent to the control GFP-expressing cells (Supplemental Figure 7A), consistent with the previous results from Verbeek et al. (2008). These findings suggest that the SCA14 mutant γPKCs are not activated by TPA, leading to impaired perinuclear translocation and a failure to induce cell shrinkage and activate macropinocytosis.

## Discussion

In the present study, we revealed that treatment of γPKC-expressing cells with TPA for 30–60 min triggers the perinuclear translocation of γPKC and its substrate p-MARCKS, eventually leading to cell shrinkage, remodeling of the actin cytoskeleton and the activation of macropinocytosis. However, SCA14 mutant γPKC does not mediate these phenomena in response to TPA treatment, as TPA fails to activate SCA14 mutant γPKC and increase the phosphorylation of MARCKS.

As previously reported for α and β PKC (Becker and Hannun, 2003), we found that treatment with a phorbol ester for more than 30 min induces the perinuclear translocation of γPKC, accompanied by cell shrinkage, in a PKC kinase activity- and microtubule-dependent manner (Figure 1). This translocation is caused by a different mechanism than its translocation to the plasma membrane, which is independent of the cytoskeleton (Sakai et al., 1997) and PKC activity (Seki et al., 2005b). PKC molecules are thought to randomly move throughout the cytosol in the resting state. PKC activators, such as diacylglycerol and TPA, can bind to the PKCs and trap them at the plasma membrane, which drives the translocation of PKC from the cytosol to the plasma membrane (Schaefer et al., 2001). In contrast, the subsequent perinuclear translocation involves active transport along the microtubule, which is triggered by PKC substrate phosphorylation.

γPKC triggers actin remodeling and activates macropinocytosis in response to TPA (Figures 3A,B). Macropinocytosis is actin-dependent endocytic pathway and is important for the morphological changes of the cells by the incorporation of membrane components (Lim and Gleeson, 2011). Indeed, it has been recently reported that macropinocytosis-mediated membrane retrieval contributes to the collapse of the growth cone in repulsive axon guidance (Kabayama et al., 2009; Kolpak et al., 2009). We also demonstrated that JosD1, a membrane-targeted deubiquitinating enzyme, regulates both changes in cell morphology and macropinocytosis (Seki et al., 2013). In the present study, the components of the plasma membrane are incorporated into these γPKC-positive vesicles (Supplemental Figure 4). Therefore, the cell shrinkage that accompanied with perinuclear translocation of γPKC would be mediated by the incorporation of membrane components via macropinocytosis.

Because the perinuclear translocation of γPKC depends on PKC kinase activity (Figures 1C,D), the phosphorylation of PKC substrates at the plasma membrane would be involved in the PKC-mediated regulation of macropinocytosis. We found that phosphorylated MARCKS is colocalized with the perinuclear translocated γPKC and F-actin in response to TPA (Figure 4A), strongly suggesting the possible involvement of MARCKS in the regulation of macropinocytosis. It has been proposed that MARCKS functions in the remodeling of the actin cytoskeleton, either through direct binding and crosslinking of F-actin (Hartwig et al., 1992) or by modulating the availability of PIP_2_ (Laux et al., 2000), which provides essential signals for the regulation of actin dynamics. Additionally, MARCKS-GFP-expressing cells exhibited large vesicles that were positive for both MARCKS-GFP and F-actin (Supplemental Figure 7B). These findings suggest that the regulation of the actin cytoskeleton by MARCKS might play an important role in the activation of macropinosomes in response to TPA.

In the present study, prolonged treatment with TPA failed to induce the perinuclear translocation of γPKC, cell shrinkage and enhanced macropinocytosis in cells expressing SCA14 mutant γPKC (S119F, S119P, and G128D) (Figures 2, 3); however, these mutants were able to translocate to the plasma membrane, as previously described (Seki et al., 2005a; Adachi et al., 2008; Verbeek et al., 2008). Because these phenomena induced by prolonged TPA treatment were dependent on PKC kinase activity, it is possible that the SCA14 mutant γPKC fails to phosphorylate its substrates in response to TPA, at least at the plasma membrane. Our group and others have reported that mutant γPKC has a higher kinase activity than the WT kinase in the absence of PKC activators (Verbeek et al., 2005; Adachi et al., 2008). However, Verbeek et al. revealed that mutant γPKC has a lower activity at the plasma membrane in response to TPA (Verbeek et al., 2008). This would be due to the decreased affinity of mutant γPKC for the PKC activator at the plasma membrane. The translocation of mutant γPKC to the membrane looks similar to that of WT. However, single-molecule imaging revealed that the residence time of mutant γPKC at the plasma membrane was significantly decreased, compared with that of WT γPKC (Adachi et al., 2008). This property of mutant γPKC would result in the decreased phosphorylation of the membrane substrates, leading to the impairment of perinuclear translocation, cell shrinkage and macropinocytosis. Indeed, we confirmed the decreased phosphorylation of MARCKS in cells expressing mutant γPKC (Supplemental Figure 7A). This deregulation of mutant PKC activity at the membrane could result in the molecular pathogenesis of SCA14. Our previous report revealed that SCA14 mutant γPKC induces the improper development of PC dendrites (Seki et al., 2009). This morphological change might be caused by impaired γPKC activity at the plasma membrane and deregulation of the actin cytoskeleton and macropinocytosis. Further studies are necessary to determine whether deregulation of actin cytoskeleton and macropinocytosis are also observed in primary cultured PCs expressing SCA14 mutant γPKC.

In summary, our present findings provide novel insight into the mechanisms of PKC-mediated macropinocytosis and suggest that the regulation of the actin cytoskeleton and macropinocytosis is related to disease pathogenesis. Macropinocytosis might be a novel target for the treatment of SCA14. It is known that macropinocytosis is inhibited by amiloride and its analogs, which are potent inhibitors of the Na^+^/H^+^ exchange protein at the plasma membrane (Swanson and Watts, 1995). Furthermore, macropinocytosis depends on actin organization and is regulated by various molecules, including PI3-kinase and many small GTPases (Lim and Gleeson, 2011). Chemicals that affect these macropinocytosis regulators would be candidate novel therapeutics for the treatment of SCA14.

### Conflict of interest statement

The authors declare that the research was conducted in the absence of any commercial or financial relationships that could be construed as a potential conflict of interest.
