# Supplementary material for: Deregulation of the actin cytoskeleton and macropinocytosis in response to phorbol ester by the mutant protein kinase C gamma that causes spinocerebellar ataxia type 14
Source: Front Physiol. 2014 Apr 1;5:126. doi: 10.3389/fphys.2014.00126 (PMC3978357; doi:10.3389/fphys.2014.00126)
Supplement: Supplementary file 1 [file Presentation1.PDF]

Supplementary Material

Deregulation of the actin cytoskeleton and macropinocytosis in response to phorbol ester by the mutant protein kinase C gamma that causes spinocerebellar ataxia type 14

Kazuhiro Yamamoto[1], Takahiro Seki[1,2]*, Hikaru Yamamoto[1,3], Naoko Adachi[3], Shigeru Tanaka[1], Izumi Hide[1], Naoaki Saito[3] and Norio Sakai[1]

[1]Department of Molecular and Pharmacological Neuroscience, Graduate School of Biomedical & Health Sciences, Hiroshima University, Hiroshima 734-8551, Japan [2]Department of ChemicoPharmacological Sciences, Graduate School of Pharmaceutical Sciences, Kumamoto University,
Kumamoto 862-0973, Japan [3]Biosignal Research Center, Kobe University, Kobe 657-8501, Japan

*Correspondence: Takahiro Seki, Ph.D., Department of Chemico-Pharmacological Sciences, Graduate School of Pharmaceutical Sciences, Kumamoto University, 5-1 Oe-Honmachi, Chuo-ku, Kumamoto 862-0973, Japan, E-mail: takaseki@kumamoto-u.ac.jp

1. Supplementary Data

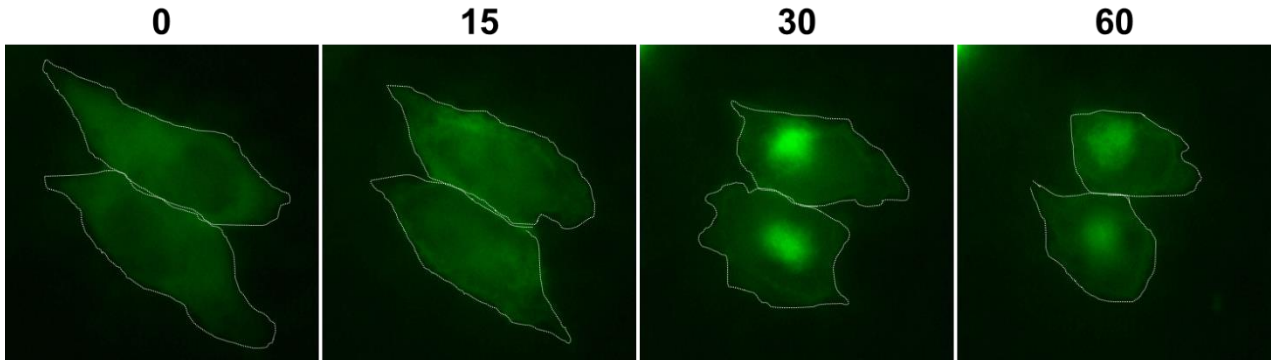

Supplemental Figure 1

Temporal changes in the size of the cells expressing γPKC-GFP treated with TPA.

The shapes of cells shown in figure 1A are highlighted by dot lines.

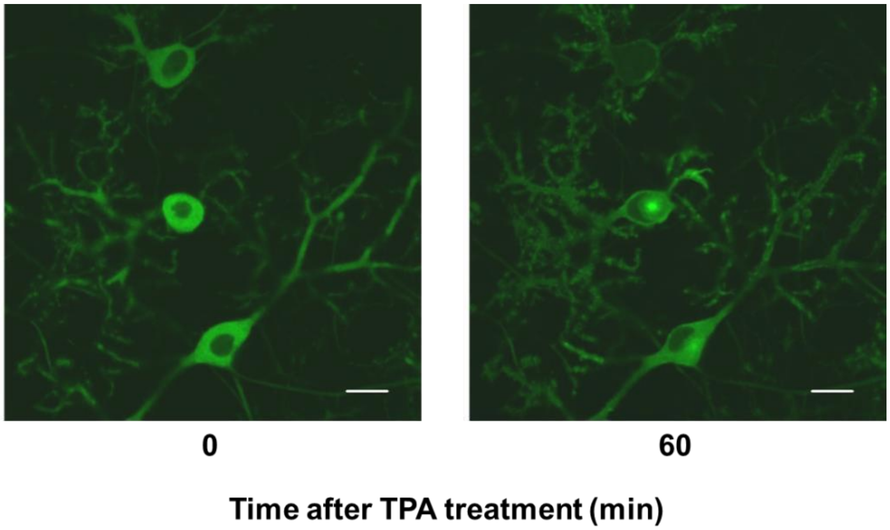

Supplemental Figure 2

Perinuclear translocation of γPKC after prolonged TPA treatment in primary cultured Purkinje cells.

Representative fluorescence images of Purkinje cells (PCs) before (0 min) and after (60 min) TPA treatment are shown. Time-lapse observation was conducted in primary-culture PCs expressing γPKC-GFP in 1-min intervals for 60 min. The perinuclear translocation of γPKC-GFP was observed 60 min after TPA treatment. On the other hand, the reduction in cell size was not remarkable in the Purkinje cells. Bar = 10 μm.

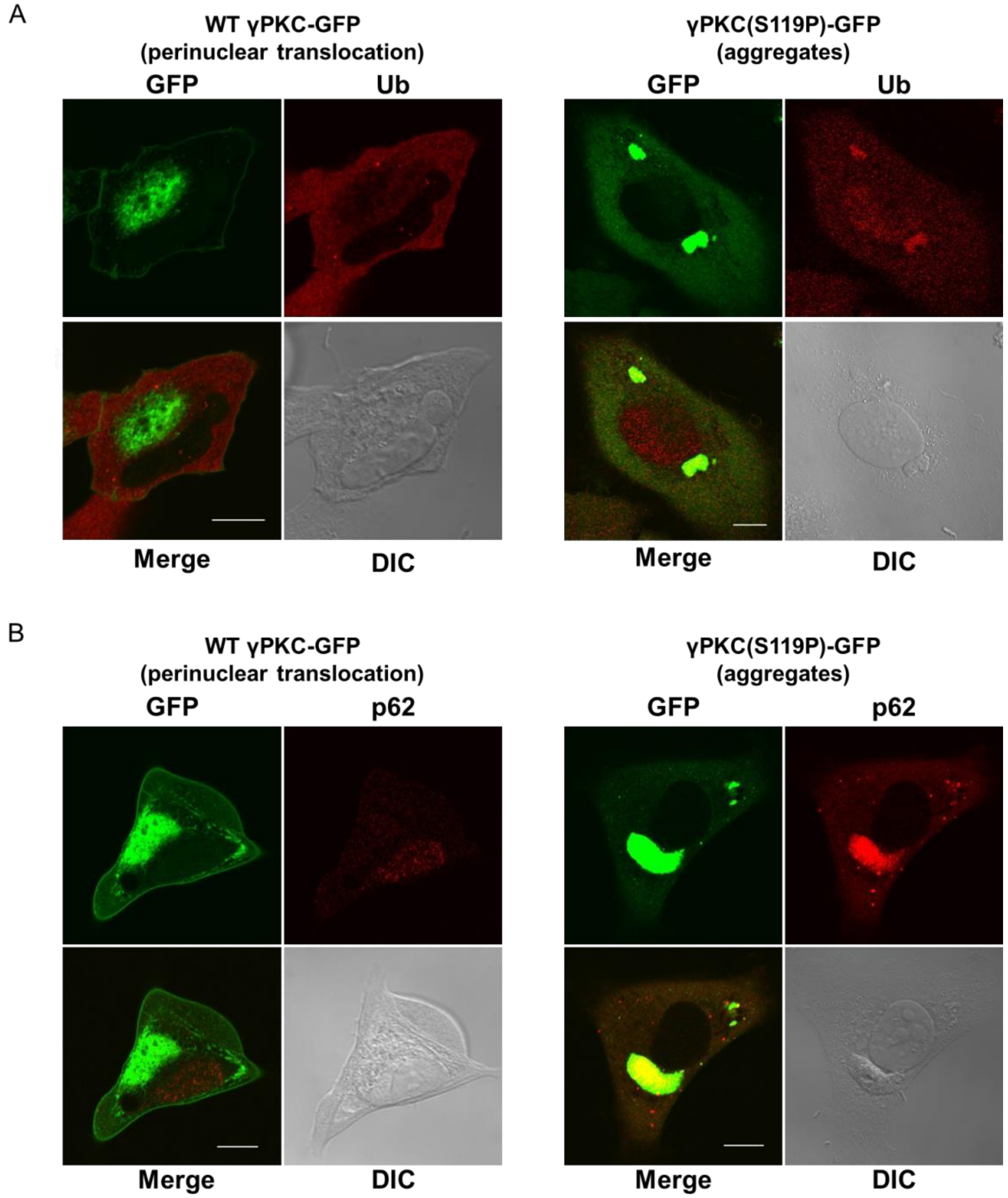

Supplemental Figure 3

Perinuclear translocated WT γPKC is different from the aggregates of mutant γPKC.

The perinuclear WT γPKC-GFP was compared with the aggregates of mutant γPKC-GFP. In this experiment, the perinuclear translocation of WT γPKC-GFP was induced by treatment with 100 nM TPA for 30 min, while TPA was not added to the HeLa cells expressing S119P γPKC-GFP (S119PGFP). **(A)** Representative images (γPKC-GFP, ubiquitin, merged and DIC) are shown. The perinuclear translocation in HeLa cells expression WT γPKC-GFP (left) and the aggregates of S119P γPKC-GFP (right) were immunostained with an anti-ubiquitin antibody. The aggregates of mutant

γPKC-GFP were positive for ubiquitin, but the perinuclear WT γPKC-GFP was negative. Scale bar = 10 μm.

(B) Representative images (γPKC-GFP, p62, merged and DIC) are shown. The perinuclear translocation of HeLa cells with WT γPKC-GFP (left) and the aggregates of S119P γPKC-GFP (right) were immunostained with an anti-p62 antibody. The aggregates of mutant γPKC-GFP were positive for p62, but the perinuclear WT γPKC-GFP was negative. Scale bar = 10 μm.

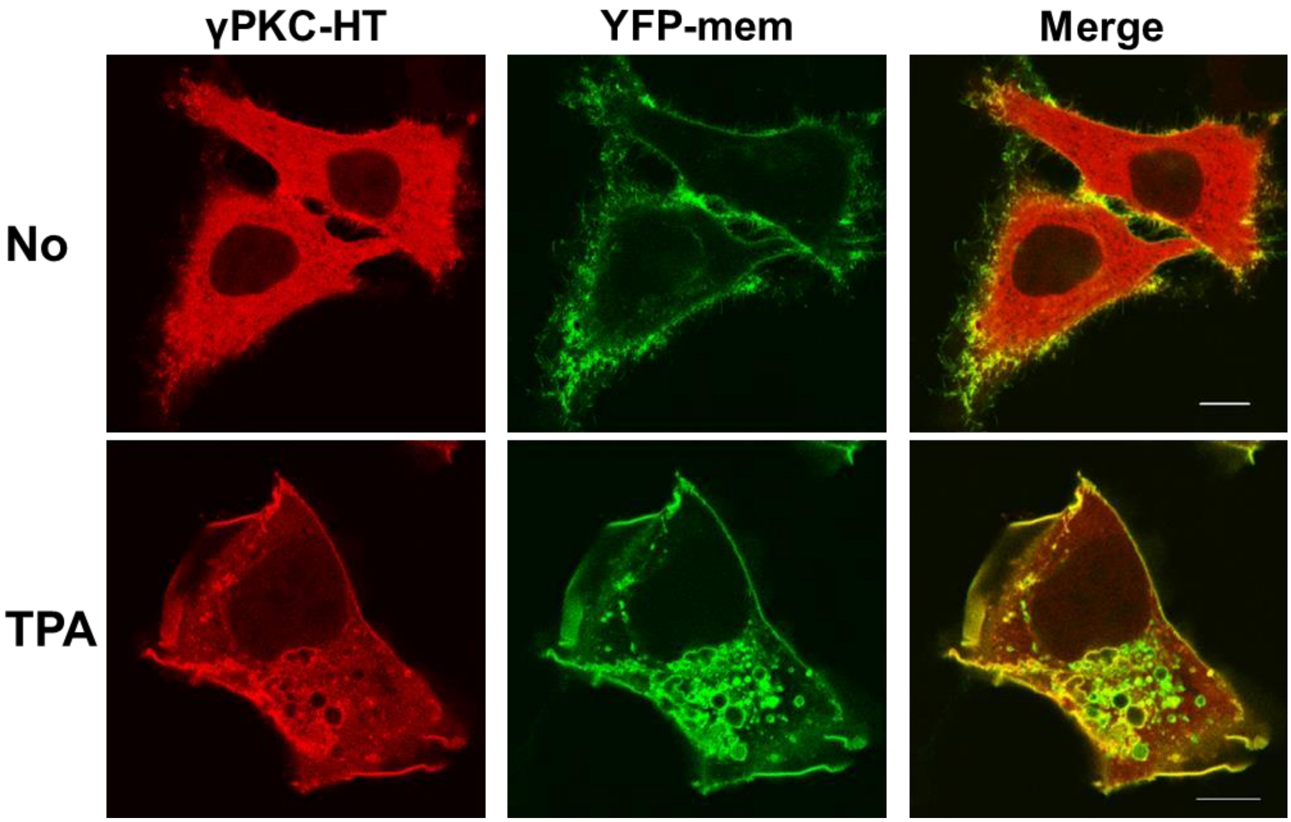

Supplemental Figure 4

TPA induces membrane incorporation in cells expressing □PKC.

Representative images (□PKC-HT, YFP-mem and merged) of cells expressing □PKC-HT and YFPmem, a membrane-bound YFP, in the absence (upper panels) or presence (lower panels) of TPA treatment for 30 min. YFP-mem was strongly colocalized with □PKC-HT around the vesicles in the TPA-treated cells. Scale bar = 10 □m.

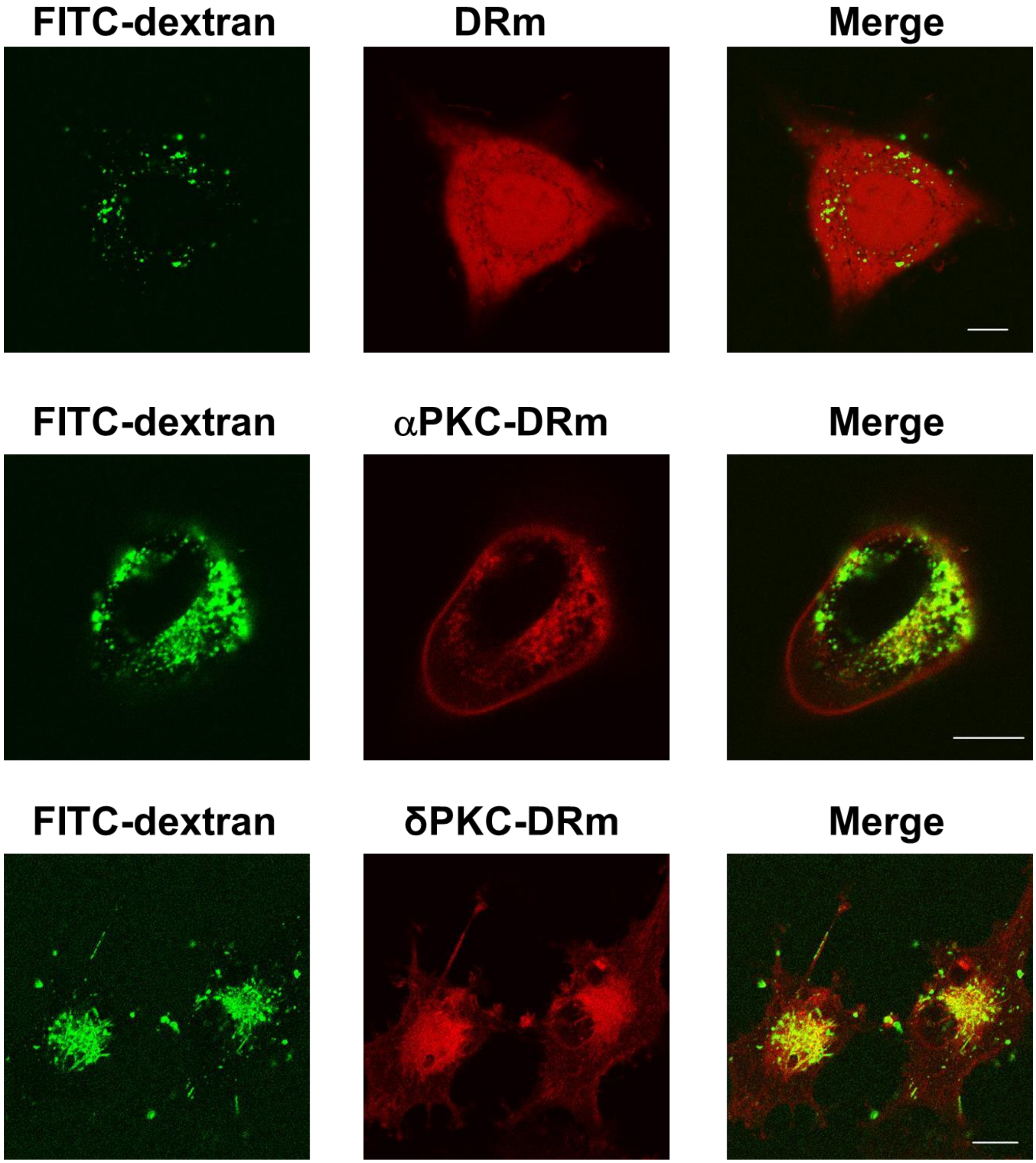

Supplemental Figure 5

□PKC and δPKC cause macropinocytosis in response to TPA treatment.

HeLa cells expressing DsRed monomer (DRm), □PKC-DRm and δPKC-DRm were incubated with FITC-dextran and 100 nM TPA for 30 min. Representative images (FITC-dextran, DRm and merged) are shown. TPA caused the perinuclear translocation of PKC-DRm and the uptake of

FITCdextran in cells expressing □PKC-DRm and δPKC-DRm, but not in cells expressing DRm alone. Scale bar = 10 µm.

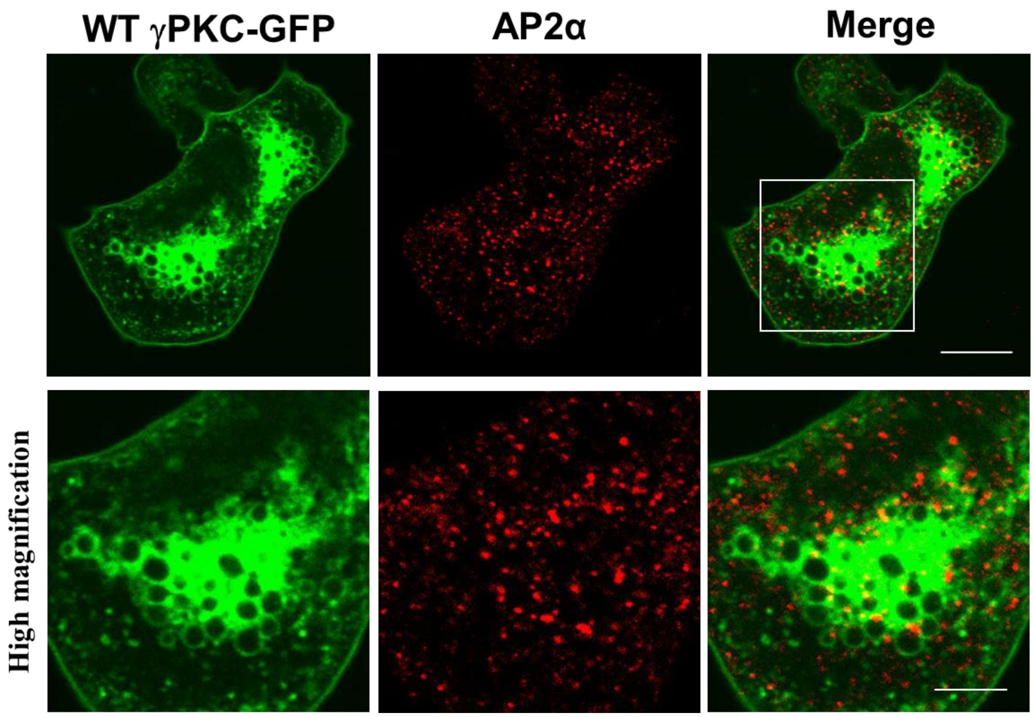

Supplemental Figure 6

Vesicles with γPKC are not clathrin-coated endosomes.

Representative images (γPKC-GFP, AP2 and merged) are shown. HeLa cells expressing WT γPKCGFP were fixed 30 min after TPA treatment and were immunostained with an anti-AP2α antibody, a marker of clathrin-coated vesicles. The lower panels show the high magnification images of the boxed area in the merged image in the upper panels. AP2α-positive vesicles were obviously small and did not colocalize with the vesicles surrounded by γPKC-GFP. The scale bars of the merged and high magnification images are 10 and 5 µm, respectively.

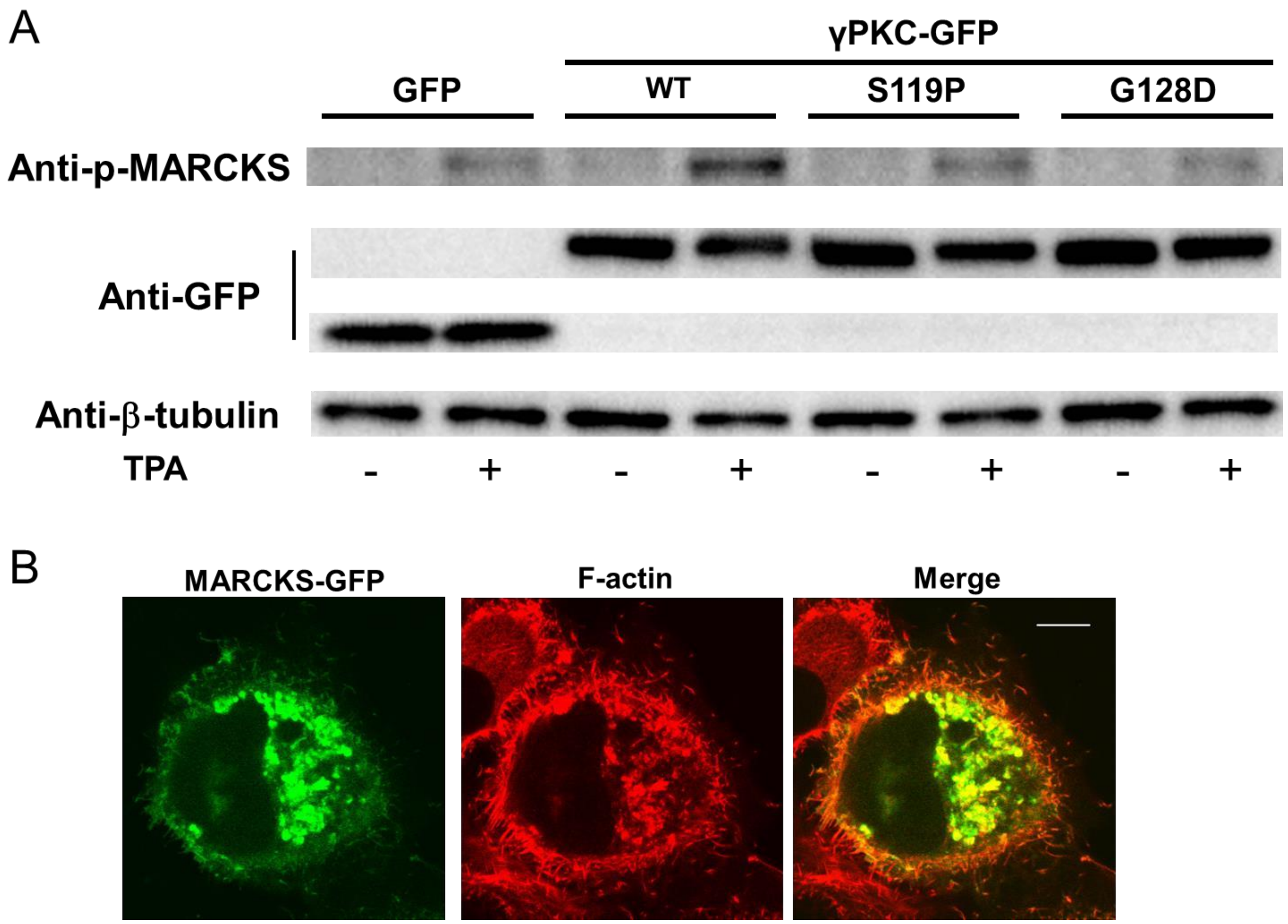

Supplemental Figure 7

Phosphorylation of MARCKS is involved in TPA-triggered macropinocytosis.

(A) Immunoblot analysis of HeLa cells expressing GFP, WT and mutant (S119P and G128D) γPKCGFP with p-MARCKS, GFP and β-tubulin antibodies in the absence or presence of 100 nM TPA treatment for 30 min. TPA prominently increased the level of p-MARCKS in cells expressing WT, compared to the cells expressing GFP alone. In contrast, the TPA-induced phosphorylation of MARCKS in cells expressing mutant γPKC-GFP was similar to that in the GFP-expressing cells.

(B) MARCKS-GFP localizes to F-actin-positive cytoplasmic vesicles. HeLa cells expressing MARCKS-GFP were fixed and stained with phalloidin-TRITC. MARCKS-GFP occasionally colocalized with F-actin in the cytoplasmic vesicles.
